# Declines in Physical Performance by Sex and Age Among Nondisabled Community-Dwelling Older Japanese During a 6-Year Period

**DOI:** 10.2188/jea.JE20100138

**Published:** 2011-05-05

**Authors:** Tatsuro Ishizaki, Taketo Furuna, Yuko Yoshida, Hajime Iwasa, Hiroyuki Shimada, Hideyo Yoshida, Shu Kumagai, Takao Suzuki

**Affiliations:** 1 Kyoto University School of Public Health, Kyoto, Japan; 2 Sapporo Medical University, Hokkaido, Japan; 3 Tokyo Metropolitan Institute of Gerontology, Tokyo, Japan; 4 University of Tokyo Graduate School of Medicine, Tokyo, Japan; 5 National Institute for Longevity Sciences, Aichi, Japan; 6 University of Human Arts and Sciences, Saitama, Japan

**Keywords:** aged, aging, hand-grip strength, walking speed, longitudinal study

## Abstract

**Background:**

Few studies have examined whether declines over time in hand-grip strength (HGS) and fast walking speed (FWS) differ by sex and age among non-Western community-dwelling older adults. This study aimed to quantify changes in HGS and FWS over the 6-year period from 1994 to 2000 and examine whether these changes differed by sex and baseline age among older individuals in a Japanese community.

**Methods:**

We conducted a community-based prospective cohort study. The participants were 513 nondisabled men and women aged 67 to 91 years at the 1994 survey. Independent variables regarding time since baseline, in addition to various time-dependent and time-independent covariates, were obtained in 1994, 1996, 1998, and 2000. The outcome variables were HGS and FWS assessed at each survey. All data on independent and dependent variables that were collected at each survey were simultaneously analyzed using a linear mixed-effects model.

**Results:**

The linear mixed-effects model revealed significant declines in both HGS (−0.70 kg/year, *P* < 0.001) and FWS (−0.027 m/sec/year, *P* < 0.001) among nondisabled older participants who had analyzable data in any survey during the 6-year period. Sex was significantly associated with the rate of decline in HGS (*P* < 0.001), but not FWS (*P* = 0.211).

**Conclusions:**

In this analysis of nondisabled older Japanese, a mixed-effects model confirmed a significant effect of aging on declines in HGS and FWS and showed that men had a significantly steeper decline in HGS than did women during a 6-year period.

## INTRODUCTION

Physical performance measures such as hand-grip strength (HGS) and walking speed are important health indicators, and have been reported to be significant predictors of disability.^1^^–^^4^ Furthermore, these measures are not just important components of frailty^5^; they are also used to forecast health care needs among older populations. Thus, it is important to identify longitudinal changes in physical performance that occur with aging.^6^^–^^9^ The precise identification of longitudinal changes in physical performance over time requires the collection of longitudinal data (with a minimum of 3 measurements for both independent and dependent variables)^9^ and a mixed-effects model that takes into account both individual patterns of change (individual random effects) and population averages (fixed effects).^10^ However, only a few studies have used more than 2 data points to evaluate age-related longitudinal changes in physical performance among community-dwelling older adults in Western countries.^11^^–^^13^ In addition, because life expectancy at birth is longer in women than in men in developed countries, changes in physical performance may differ by sex and age. Nevertheless, few studies have elucidated whether declines in physical performance over time differ by sex and baseline age.^12^ The Japanese have the highest life expectancy at birth in the world,^14^ making them an ideal population for studying age-related changes in physical performance. In this study, we analyzed repeatedly collected data using a mixed-effects model that accounts for inter-individual variations in aging so as to quantify declines in HGS and fast walking speed (FWS) over a 6-year period in community-dwelling older Japanese. In addition, we examined whether those declines varied by sex and baseline age.

## METHODS

### Data source and study participants

The source of data for this study was the Tokyo Metropolitan Institute of Gerontology-Longitudinal Interdisciplinary Study on Aging (TMIG-LISA),^4^^,^^15^^,^^16^ which surveyed older individuals from Nangai Village, a rural community in the northern Japanese prefecture of Akita.^17^ Nangai Village is a mainly agricultural area in Akita Prefecture in northern Honshu, which is 1 of the 4 main islands of Japan. The total population of the village in 1992 was 5136. In June 1992, before the baseline survey, a questionnaire was used to assess the mobility of all residents of the village aged 65 years or older (*n* = 940). Residents with the ability to go out in the neighborhood or to use public transportation were regarded as ambulatory. As a result of the June pre-survey, 852 people were identified as ambulatory residents and were asked to participate in the baseline survey held from July to August 1992. A total of 748 ambulatory residents aged 65 or older participated in the 1992 survey (participant rate = 85%). For the present study, we limited the original pool of participants to those individuals who participated in at least 1 follow-up survey conducted at organized gatherings at municipal community centers in 1994, 1996, 1998, and 2000. We did not use data collected in the 1992 survey because we used a different method of HGS assessment at that time point only. Furthermore, we only included nondisabled individuals—defined as those reported to be independent in both activities of daily living (ADL) and instrumental activities of daily living (IADL) in the 1994 survey—to account for the natural process of age-related disablement in functioning. Capacity for ADL was measured using 4 items: walking, feeding, bathing, and dressing.^4^ An individual was defined as ADL-dependent if they were not independent in any item of ADL. Capacity for IADL was measured using the 5-item instrumental self-maintenance subscale of the TMIG Index of Competence, which was developed to assess levels of functional competence greater than those required for ADL in older people.^4^^,^^18^ The index is further subdivided into 3 subscales of functional competence, which are termed instrumental self-maintenance, intellectual activity, and social role. IADL-dependence was defined as a loss of independence in any of the 5 items of the instrumental self-maintenance subscale of the TMIG Index of Competence, namely, being able to use public transportation by oneself, being able to shop for daily necessities, being able to prepare meals by oneself, being able to pay bills, or being able to manage one’s own bank account. This study was approved by the Institutional Review Board of the TMIG.

### Assessment of physical performance and collection of data for independent variables

We conducted a longitudinal evaluation of physical performance and other variables, using data obtained from the surveys in 1994, 1996, 1998, and 2000. At each survey, we measured HGS in the dominant hand using a Smedley-type hand dynamometer. To assess FWS, we asked participants to walk at their maximum speed on a straight, flat, 11-meter-long walkway. FWS was measured over the 5-meter distance from 3 meters to 8 meters from the start of the walkway. Reproducibility of the walking test has been reported previously.^19^ The assessment methods used in this study remained consistent throughout the study period.

We developed a comprehensive analytical model that includes time (i.e. the number of years since the 1994 survey), time-invariant independent variables, including 2 demographic variables (sex and age in the year 1994), and time-dependent variables, which comprise 5 physical variables (body mass index [BMI], chronic medical condition, complaint of pain, history of hospitalization during the past year, and serum albumin concentration), 2 psychological variables (depressive symptomatology and intellectual activity), and 2 lifestyle variables (current smoking status and current level of alcohol consumption).

All data for the independent variables used in this study, except BMI (kg/m^2^) and serum albumin concentration, were obtained from the interview at each survey. Height and body weight were measured at the survey site. BMI was calculated as body weight (kg) divided by height squared (m^2^). Participants with any history of hypertension, diabetes, or heart disease were categorized as having a chronic medical condition. We defined a complaint of pain as the presence of any pain at the time of the interview. Depressive symptomatology was measured using the Japanese version of the Geriatric Depression Scale (GDS)^20^^,^^21^ in the 1994 survey. We considered a GDS score of at least 11 points to indicate depressive symptomatology (range: 0–30). Since 1996, an abbreviated version of the GDS has been used,^22^ and depressive symptomatology was defined as a score of 5 points or higher (range: 0–15). Intellectual activity was assessed using the subscales in the TMIG Index of Competence.^4^^,^^18^ The subscale consists of 4 dichotomized items. Poor intellectual activity was defined as a loss of activity in any item of the intellectual activity subscale in the index. The items were: being able to fill out forms for one’s pension, reading newspapers, reading books or magazines, or being interested in news stories or issues dealing with health. As for the lifestyle-related variables, answer options for current alcohol drinking status and current smoking status were “yes,” “quit,” and “never.” At each survey, we collected a non-fasting blood sample from the antecubital vein for routine hematological and biochemical tests while the participant was sitting. The blood sample was sent via air carrier to a single commercial laboratory (Special Reference Laboratories, Inc., Tokyo, Japan). Serum albumin levels were enzymatically determined by a sequential autoanalyzer (HITACHI 736, Hitachi, Ltd.). For these measurements, the coefficients of variation of internal quality controls were less than 1.0%. The methods used for the above measurements remained consistent throughout the study period.

### Statistical analysis

We performed statistical analyses using SAS Version 9.2 (SAS Institute, Inc., Cary, NC, USA). First, basic participant characteristics from the 1994 survey were classified by sex and age group, using mean, standard deviation, and range (minimum and maximum values) for continuous variables and frequency (%) for categorical variables.

Rates of change in HGS and FWS were determined for each 2-year interval (i.e. between 1994 and 1996, 1996 and 1998, and 1998 and 2000) and categorized into 4 groups: ≥10% increase, <10% change, ≥10% to <20% decrease, and ≥20% decrease. Rate of change in physical performance was calculated as (value*_time_*_+2_ − value*_time_*)/value*_time_* × 100%, where *_time_* is the number of years since the 1994 survey. We then calculated the proportion of participants in each of the 4 groups at the beginning and end of each 2-year interval.

Changes in physical performance across all 4 surveys, as well as whether these changes over time varied by sex or baseline age, were analyzed by fitting individual growth models^23^ using the linear mixed-effects model (PROC MIXED in SAS) with random intercepts and random slopes for time. This method was used because we wanted to allow both intercepts and slopes to vary across participants. We used Kenward–Roger correction to estimate robust standard errors in the analyses.^24^ The linear mixed-effects model assumes that the random intercepts and random slopes for time are normally distributed. The within-person residual covariance matrix was evaluated and modeled with the unstructured correlation structure. This statistical analysis accounted for all available data points from the 4 surveys during the 6-year period—so that participants with incomplete data sets were not excluded from the analysis—and for multiple observations per participant that were likely to be correlated. In the linear mixed-effects model used in this study, the dependent variable was either HGS or FWS. Each model included time, which represented the number of years since the 1994 survey, time-invariant independent variables such as sex and baseline age (for which age 67 years was set at 0), and 2 interaction terms such as sex × time and time × baseline age. In addition to these variables, the 9 time-dependent independent variables described above (5 physical variables, 2 psychological variables, and 2 lifestyle variables) were included in the analytical models. The coefficients of time represented the average rates of change in physical performance over time,^10^ and coefficients of time interaction terms with either sex or baseline age represented whether time slope varied by sex or baseline age. Furthermore, coefficients of independent variables, except time and the 2 time interaction terms, represented the change in a value from baseline. Finally, a sensitivity analysis was performed to examine the impact of selective attrition bias on results from the primary analyses by using the same models for data from participants who had data at 4 data points, without missing measurements of any dependent or independent variable used in the primary analyses. All reported *P* values are 2-tailed, and a value of *P* less than 0.05 was considered statistically significant.

## RESULTS

Among the 712 individuals who participated in the 1994 survey, a total of 513 nondisabled participants were eligible as the cohort for our 6-year follow-up period (mean age, 72.3 years; standard deviation, 4.5; range, 67–91 years; men = 44.8%). We excluded 194 disabled participants in the 1994 survey (ADL-dependent only = 4, IADL-dependent only = 149, ADL- and IADL-dependent = 41) and 5 participants with missing measurements for ADL and IADL status. Over the 4 surveys during the 6-year period, 1518 and 1465 analyzable observations were collected for HGS and FWS, respectively, among the 513 nondisabled participants in 1994 (Table 1). The average numbers of HGS and FWS data points were 3.0 and 2.9, respectively, and approximately 90% of participants completed physical performance measurements at least twice during the 6-year period. A total of 227 and 195 participants had analyzable data for HGS and FWS, respectively, for all 4 follow-up time points and no missing data for any independent variable. Table 2 shows the baseline characteristics of the study participants from the 1994 survey. Mean baseline HGS and FWS values for men were greater than those for women in the same age group, and these values tended to decrease in older age groups. Table 3 shows variations in the rates of change in HGS and FWS at the beginning and end of each 2-year interval during the 6-year follow-up period. More than 60% of participants exhibited a decrease of 10% or more in physical performance during all 2-year intervals, except for HGS in men between 1996 and 1998.

**Table 1.** Participants in follow-up surveys of the Tokyo Metropolitan Institute of Gerontology-Longitudinal Study on Aging (TMIG-LISA), 1994–2000

	Survey year			
	
	1994	1996	1998	2000
Eligible participants^a^	513	469	434	407
**Participants excluded from data analysis**				
Participants at home visit survey	—	9	12	11
Hospitalized or institutionalized	—	13	12	7
Nonrespondents	—	3	4	1
Participants who died during ​ follow-up	—	19	32	36
**Cumulative number of analyzable** **observations at each survey**				
Hand-grip strength	477	884	1224	1518
Fast walking speed	471	874	1183	1465

^a^The cohort of this study includes 513 participants who were independent in activities of daily living and instrumental activities of daily living in the 1994 survey and participated in the survey conducted through organized gatherings at municipal community centers.

**Table 2.** Baseline characteristics of nondisabled participants in the Tokyo Metropolitan Institute of Gerontology-Longitudinal Study on Aging (TMIG-LISA) in 1994 (*n* = 513)^a^

		Men			Women		
			
	Age group	67–69	70–74	75+	67–69	70–74	75+
Body mass index ​ (kg/m^2^)	*n*	*72*	*85*	*57*	*96*	*104*	*69*
	Mean	22.2	21.7	21.9	23.4	23.4	22.9
	(SD)	(2.8)	(2.8)	(2.9)	(3.7)	(3.8)	(3.0)
	Range	16.8–29.6	15.7–32.0	17.4–28.8	15.0–32.3	15.8–35.1	16.0–29.3

Chronic medical ​ condition^b^	*n*	*78*	*88*	*63*	*98*	*111*	*74*
	% present	9.0	10.2	6.4	12.2	9.0	13.5

Complaint of pain	*n*	*79*	*88*	*63*	*98*	*111*	*74*
	% yes	53.2	47.7	52.4	67.4	80.2	68.9

History of ​ hospitalization during ​ the past year	*n*	*79*	*88*	*63*	*98*	*111*	*74*
	% yes	2.5	4.6	11.1	10.2	14.4	14.9

Depressive ​ symptomatology	*n*	*77*	*88*	*63*	*98*	*110*	*74*
	% present	24.7	23.9	28.6	25.5	33.6	25.7

Intellectual activity	*n*	*79*	*88*	*63*	*98*	*111*	*74*
	% poor	44.3	35.2	38.1	41.8	55.9	54.1

Current alcohol ​ consumption	*n*	*79*	*88*	*63*	*98*	*111*	*74*
	% yes	63.3	69.3	54.0	24.5	16.2	21.6

Current smoking habit	*n*	*79*	*88*	*63*	*98*	*111*	*74*
	% yes	39.2	39.8	30.2	1.0	0.9	0.0

Serum albumin (g/L)	*n*	*72*	*85*	*57*	*94*	*104*	*69*
	Mean	41.0	40.7	40.0	42.2	41.8	41.6
	(SD)	(2.3)	(2.3)	(1.9)	(2.2)	(2.3)	(2.4)
	Range	34–47	34–47	35–44	34–47	37–47	32–48

Hand-grip strength (kg)	*n*	*72*	*85*	*56*	*95*	*103*	*69*
	Mean	34.4	33.4	30.8	23.2	22.2	19.9
	(SD)	(6.7)	(5.7)	(5.4)	(4.0)	(4.3)	(5.4)
	Range	9–47	20–51	19–42	11–32	12–32	5–31

Fast walking speed ​ (m/sec)	*n*	*71*	*84*	*55*	*94*	*102*	*68*
	Mean	2.08	2.00	1.85	1.82	1.61	1.48
	(SD)	(0.41)	(0.42)	(0.35)	(0.32)	(0.34)	(0.42)
	Range	1.19–3.13	0.29–2.94	1.19–2.78	0.61–2.50	0.63–2.78	0.38–2.63

^a^The cohort comprised participants who were independent in activities of daily living and instrumental activities of daily living in the 1994 survey and participated in the survey conducted through organized gatherings at municipal community centers.

^b^Defined as a history of hypertension, diabetes, or heart disease.

SD = standard deviation.

**Table 3.** Differences in rates of change in hand-grip strength and fast walking speed for each 2-year interval during the 6-year follow-up period

	Baseline (1994) to 1996		1996 to 1998		1998 to 2000	
**Hand-grip strength**	Men	Women	Men	Women	Men	Women
	*n* = 161	*n* = 231	*n* = 124	*n* = 185	*n* = 105	*n* = 155
Rate of change between 2 time points^a^	%	%	%	%	%	%
≥10% increase	4.3	14.3	24.2	28.1	8.6	11.0
<10% change	16.1	17.3	42.7	30.8	17.1	18.7
≥10% to <20% decrease	62.1	54.1	31.5	35.1	62.9	45.8
≥20% decrease	17.4	14.3	1.6	5.9	11.4	24.5

**Fast walking speed**	Men	Women	Men	Women	Men	Women
	*n* = 156	*n* = 227	*n* = 106	*n* = 168	*n* = 84	*n* = 139
Rate of change between 2 time points^a^	%	%	%	%	%	%
≥10% increase	7.7	14.5	7.5	10.1	15.5	11.5
<10% change	28.8	26.0	13.2	14.9	22.6	23.7
≥10% to <20% decrease	55.8	50.7	56.6	53.0	51.2	51.8
≥20% decrease	7.7	8.8	22.6	22.0	10.7	12.9

^a^Rate of change during a 2-year interval was calculated as (value*_time_*_+2_ − value*_time_*)/value*_time_* × 100 (%), where *_time_* is the number of years since the 1994 survey.

Table 4 shows that, on average, both HGS and FWS significantly and linearly declined during the 6-year period. The mean decreases in HGS and FWS were −0.70 kg/year (*P* < 0.001) and −0.027 m/sec/year (*P* < 0.001), respectively. Sex was significantly associated with the rate of decline in HGS (*P* < 0.001), but not FWS (*P* = 0.211); however, baseline age was not significantly associated with the rate of decline in HGS or FWS. Variations in intercepts in both HGS and FWS and in the rate of FWS decline were significantly prevalent among participants in this longitudinal study (*P* < 0.001), even though inter-individual variation was accounted for in the mixed-effects model. In the sensitivity analyses, the 95% confidence intervals for the slopes of decline in both HGS and FWS overlapped with those in the primary analysis (data not shown). The sensitivity analyses also revealed that sex was associated with rate of decline in HGS, but not in FWS, and that intercepts for both HGS and FWS significantly varied among participants who had data for all 4 data points and no missing measurements for any dependent or independent variable.

**Table 4.** Change in hand-grip strength and fast walking speed among community-dwelling older Japanese assessed at 1 or more follow-up surveys during the 6-year period from 1994 to 2000: Results from individual growth models by the linear mixed-effects model for repeated measurements

	Hand-grip strength (kg) (*n* = 500)				Fast walking speed (m/sec) (*n* = 497)			
	(Number of total analyzable observations = 1518)				(Number of total analyzable observations = 1465)			
**Fixed effect**	Estimate	95% confidence interval		*P* value	Estimate	95% confidence interval		*P* value
Intercept	10.51	5.67	15.35	<0.001	1.545	1.189	1.900	<0.001
Time (1 year since baseline)	−0.70	−0.84	−0.57	<0.001	−0.027	−0.037	−0.016	<0.001
Sex (male)	10.93	9.84	12.02	<0.001	0.320	0.238	0.402	<0.001
Age at baseline (age 67 years = 0)	−0.33	−0.43	−0.23	<0.001	−0.026	−0.033	−0.018	<0.001
Time × sex	−0.27	−0.41	−0.12	<0.001	−0.008	−0.019	0.004	0.211
Time × baseline age	−0.02	−0.03	0.002	0.076	−0.001	−0.002	0.001	0.248
Body mass index (1 kg/m^2^)^a^	0.15	0.05	0.26	0.005	−0.008	−0.015	0.0003	0.056
Chronic medical condition (absent)^a^	0.29	−0.16	0.73	0.205	−0.032	−0.064	−0.0002	0.049
Complaint of pain (absent)^a^	0.01	−0.39	0.41	0.956	−0.035	−0.064	−0.006	0.017
History of hospitalization (absent)^a^	0.42	−0.19	1.03	0.180	0.041	−0.003	0.084	0.069
Serum albumin (1 g/L)^a^	0.20	0.10	0.30	<0.001	0.008	0.001	0.015	0.030
Depressive symptomatology (absent)^a^	0.65	0.22	1.08	0.003	0.064	0.033	0.094	<0.001
Intellectual activity (good)^a^	0.31	−0.11	0.72	0.150	0.031	0.001	0.060	0.043
Current alcohol consumption (yes)^a,b^	0.96	0.30	1.63	0.005	0.032	−0.015	0.080	0.184
(quit)^a,b^	0.17	−0.71	1.05	0.707	−0.048	−0.111	0.016	0.144
Current smoking habit (yes)^a,b^	1.08	−0.08	2.23	0.068	−0.025	−0.111	0.060	0.563
(quit)^a,b^	0.60	−0.30	1.51	0.192	−0.012	−0.079	0.054	0.719

**Random effect**	Estimate	Standard error		*P* value	Estimate	Standard error		*P* value
Variance in intercept	17.22	1.52		<0.001	0.106	0.009		<0.001
Variance in time slope	0.07	0.05		0.069	0.001	0.0002		<0.001

^a^Time-dependent independent variable with potential to change over time.

^b^As compared with never drinkers or never smokers, respectively.

## DISCUSSION

In the present study of nondisabled community-dwelling older Japanese, we used a linear mixed-effects model to quantify declines in HGS and FWS using data obtained at 4 time points during a 6-year period and examined whether these declines over time varied by sex and baseline age, while taking into account inter-individual variations in aging. The results indicated that, on average, both HGS and FWS significantly and linearly declined over time. Furthermore, although men had a significantly steeper decline than women in HGS, but not in FWS, baseline age did not significantly affect the rate of decline in HGS or FWS. Both intercepts in HGS and FWS and rate of decline in FWS varied significantly among participants in this longitudinal study, even when inter-individual variation was account for in the mixed-effects model.

The most important result of this study is that the mixed-effects model showed significant average declines over a 6-year period in both HGS (−0.70 kg/year) and FWS (−0.027 m/sec/year) in a community sample of nondisabled older Japanese. Despite differences in the physical composition of older Japanese and Western adults, the average rate of HGS decline in the present study is consistent with the results of previous longitudinal studies, which were conducted mostly in Western countries.^11^^–^^13^ However, to the best of our knowledge, no study of a non-Western nondisabled community-dwelling older population has revealed statistically significant declines in both HGS and FWS using a linear mixed-effects model with repeated measurements observed more than twice.

The use of a linear mixed-effects model with repeated measurements has certain advantages in precisely estimating average rates of change in HGS and FWS during follow-up. Results from crude analyses (Table 3) show that changes in HGS and FWS between 1996 and 1998 were very different from those in other time intervals, although we used the same methods to measure physical performance at the 4 time points. It is plausible that period effects may have contributed to this variation, as it is well known that a crude comparison between 2 measurements at 2 time points does not reveal the precise rate of change in given measurements over time, because of aging and period effects. Therefore, we recommend that researchers use a mixed-effects model for repeated measurements so as to precisely identify the rate of changes in physical performance over time.

In addition to the general decline over time, we found that the slope of the decline in HGS was significantly steeper in men than in women, although men initially had greater HGS (Table 4). Several studies have reported relevant findings. The Baltimore Longitudinal Study on Aging also noted that the rates of strength decline in the upper extremities were greater in men than in women.^6^^,^^25^ Goodpaster et al. found that loss of knee extensor strength in men over a 3-year period was almost double that of women and that loss of strength in men was much greater than the concomitant loss of muscle mass, suggesting a decline in the quality of muscle.^26^ Furthermore, age-related atrophy in femoral muscle has been shown to be mainly due to loss of type II, but not type I, fiber size.^27^^,^^28^ The mean proportion of type I fiber in muscle is reported to be lower in men than in women, whereas the mean cross-sectional area of all fiber types was smaller in women than in men.^29^ These studies suggest that the difference in muscle fiber type between men and women plays a role in sex-related variation in muscle loss. However, it has also been suggested that sex differences in muscle loss are related to catabolic cytokines and anabolic hormonal factors.^30^ Further studies should examine whether cytokines and hormonal factors are independently associated with differences in the rate of change in muscle strength in community-dwelling older Japanese men and women.

This study also identified factors associated with baseline values (i.e. intercepts) of physical performance status. Male sex, absence of depressive symptomatology, and high serum albumin concentration were significantly associated with good performance in both HGS and FWS. Previous studies revealed that serum albumin concentration^31^ was significantly associated with changes in HGS and that factors associated with decline in walking speed were joint pain, weakness in the quadriceps,^32^ and depressive status.^33^ Despite major differences in the basic characteristics of our study participants, our results are consistent with those of some previous studies conducted in Western countries. Although we did not explore the mechanisms underlying the changes observed in this study, it is plausible that age-related loss of muscle mass, i.e. sarcopenia,^30^ contributed to the declines in HGS and FWS.

The major strength of this study was the use of a linear mixed-effects model with repeated measurements of the targeted samples, which allowed for variation in both longitudinal rates of change in physical performance and intercepts.^9^^,^^10^^,^^23^^,^^24^ To identify longitudinal changes in physical performance over 6 years, we used a multiple longitudinal design and performed 4 data collections between 1994 and 2000. There were an average of approximately 3 data points for both HGS and FWS during the 6-year period. This design enabled measurement of the association between physical performance and aging, while controlling for both time period and cohort effects.^10^ Therefore, we believe that this is the most appropriate method for exploring average declines in physical performance over time, while allowing for comparisons between individuals of different sex and varying age.

We cannot exclude the possibility that the results of this study are biased due to selective attrition. Individuals who participated in all surveys were likely to have better health than those who did not: the former were significantly less likely to be ADL-dependent, to be IADL-dependent, and to have a history of hospitalization. Moreover, they were an average of 1.5 years younger than participants who had both analyzable data and missing responses during the 6-year period (data not shown). However, we believe that the impact of such selective attrition bias on the results of this study was small. First, 91% to 95% of the individuals enrolled in the study participated in each survey. Second, in order to examine the magnitude of selective attrition bias in the study results, we performed supplementary sensitivity analyses with the same models used in the primary analyses and compared these results with those from the primary analyses. In the primary and supplementary analyses, the 95% confidence intervals for the adjusted coefficients of the slopes for both HGS and FWS overlapped (data not shown); selective attrition bias would be expected to result in greater adjusted coefficients with reduced participation in the surveys. These results indicate that these adjusted values for the aging effect were not significantly biased due to selective loss of participants during follow-up. Finally, participants with missing HGS or FWS measurements were more likely to have a history of hospitalization during the past year in all surveys (data not shown, *P* < 0.001). If the effect of aging on the decline in physical performance was also observed among missing, ADL-dependent, or IADL-dependent participants, or among participants with a history of hospitalization, selection bias would have increased the coefficient for the effect of aging on physical performance.

In conclusion, analysis using a mixed-effects model confirmed significant longitudinal declines in both HGS and FWS among community-dwelling, nondisabled, older Japanese during a 6-year period. Although differences in the body composition of nondisabled older Japanese and Western adults may affect objective measurement of physical performance, this study provides valuable reference values for future studies of changes in the physical performance of older populations.

## APPENDIX

### Tokyo Metropolitan Institute of Gerontology-Longitudinal Interdisciplinary Study on Aging (TMIG-LISA) Research Group

The TMIG-LISA is principally managed by the Tokyo Metropolitan Institute of Gerontology, Tokyo, Japan. The members of the TMIG-LISA Research Group who contributed to this study were: Hiroshi Shibata, Takao Suzuki (Former Principal Investigators); Shoji Shinkai (Project Leader); Hidenori Amano, Yukitoshi Aoyagi, Yoshinori Fujiwara, Taketo Furuna, Hiroshi Haga, Ken Hashizume, Hirohiko Hirano, Xyuing Hu, Kazunari Ihara, Naoyoshi Ishiyama, Tatsuro Ishizaki, Hajime Itoh, Hajime Iwasa, Masao Kanamori, Kazunori Kikuchi, Hunkyung Kim, Takashi Kinugasa, Shu Kumagai, Masayoshi Makino, Hiroshi Nagasaki, Ikuo Nasu, Naoakira Niino, Satoshi Nishizawa, Shuichi Obuchi, Hideyuki Okuzumi, Hisao Osada, Seizou Sakihara, Noboru Sekiya, Kimiko Shibasaki, Masaya Shimmei, Miho Sugiura, Yasuo Suyama, Kazuo Suzuki, Ikuma Watanabe, Shuichiro Watanabe, Masatsune Yamaguchi, Seiji Yasumura, Hideyo Yoshida, Yuko Yoshida, and Harumi Yukawa.
